# Compartment-Specific CD8 Infiltration and Disease-Specific Survival in Resected Ampullary Adenocarcinoma

**DOI:** 10.1158/2767-9764.CRC-26-0302

**Published:** 2026-09-30

**Authors:** Steve E. Kalloger, Christine Chow, Dongxia Gao, Katy Milne, James T. Topham, Joanna M. Karasinska, Brad H. Nelson, Daniel J. Renouf, John Aird, David F. Schaeffer

**Affiliations:** 1 Department of Pathology and Laboratory Medicine, University of British Columbia, Vancouver, Canada.; 2 Pancreas Centre BC, Vancouver, Canada.; 3 School of Population and Public Health, University of British Columbia, Vancouver, Canada.; 4 Molecular and Advanced Pathology Core, University of British Columbia, Vancouver, Canada.; 5 Molecular and Cellular Immunology Core, Deeley Research Centre, Victoria, Canada.; 6 Division of Medical Oncology, BC Cancer, Vancouver, Canada.; 7 Department of Medicine, University of British Columbia, Vancouver, Canada.; 8 Dept of Cellular Pathology, Mater Misericordiae University Hospital, Dublin, Ireland.; 9 Division of Anatomic Pathology, Vancouver General Hospital, Vancouver, Canada.

## Abstract

Ampullary adenocarcinoma is rare, and its immune biomarker landscape is incompletely characterized. This article evaluated mismatch repair (MMR), programmed cell death ligand 1 (PD-L1; CD274), and compartment-specific CD3 (CD3E)/CD8 (CD8A) tumor-infiltrating lymphocytes (TIL) in resected tumors and explored associations with disease-specific survival (DSS). Ninety-nine patients underwent pancreaticoduodenectomy at Vancouver General Hospital (VGH) between 1984 and 2013. Duplicate 0.6-mm tissue microarrays (TMA) were assessed for MMR proteins, PD-L1 combined positive score (CPS), and stromal and intraepithelial CD3 and CD8 counts. DSS was estimated using the Kaplan–Meier method. Exploratory Cox models were adjusted for age, histologic subtype, pathologic tumor (pT) group, pathologic node (pN) status, and adjuvant chemotherapy. There were 44 DSS events. MMR deficiency (dMMR) was present in 37 of 99 (37.4%) tumors. PD-L1 was evaluable in 87 tumors; 57 of 87 (65.5%) tumors had CPS ≥1, and 24 of 87 (27.6%) tumors had CPS ≥10. Intraepithelial CD8^+^ T cells were present in 23 of 98 (23.5%) tumors. PD-L1 CPS ≥1, PD-L1 CPS ≥10, and MMR status were not associated with DSS by log-rank testing (*P* = 0.35, *P* = 0.98, and *P* = 0.74, respectively). In the pT-adjusted model, intraepithelial CD8 presence was associated with lower disease-specific hazard [hazard ratio (HR), 0.422; 95% confidence interval (CI), 0.144–0.994; likelihood-ratio *P* = 0.048], although the Wald sensitivity test was not significant (*P* = 0.074). Continuous log-transformed intraepithelial CD8 counts showed a concordant, method-dependent signal. dMMR and PD-L1 expression were common in this historical resected cohort. Intraepithelial CD8 infiltration was associated with DSS, but the borderline and inferential method–dependent estimates require external validation. These prognostic data do not establish benefit from immune checkpoint blockade.

**Significance:**

Ampullary cancer is a rare disease in which treatment is extrapolated from other gastrointestinal cancers. In this cohort, immune features varied among tumors with functioning MMR. CD8^+^ T cells within the tumor epithelium showed an exploratory association with improved DSS, which supports the prospective evaluation of immune stratification, not immediate treatment selection.

## Introduction

The ampulla of Vater is an anatomic structure located at the junction of the pancreatic and common bile ducts. Carcinomas arising at this site are rare, with an age-adjusted incidence of approximately 0.5 to 0.6 per 100,000 in population-based studies from the United States and Europe (1, 2). Ampullary carcinomas typically involve the duodenum and the pancreatic head and are usually managed with pancreaticoduodenectomy when tumors are deemed to be resectable (3, 4). Lymph node metastases are common in resected ampullary carcinoma, and overall survival remains poor despite surgical resection (5).

Adjuvant systemic therapy is commonly considered after resection of ampullary carcinoma, but regimen selection is supported largely by periampullary evidence, pancreatic cancer trials, and expert consensus guidance (6–8). Histopathologic subtype strongly influences prognosis; patients with pancreatobiliary (PB) differentiation have consistently demonstrated inferior survival relative to those with intestinal type (9, 10). Retrospective multicenter data suggest that any benefit from gemcitabine-based adjuvant chemotherapy may be concentrated in PB/mixed rather than intestinal tumors (11).

Immune checkpoint inhibitors have become a standard targeted therapy across multiple solid tumors (12). Predictive biomarkers such as mismatch repair deficiency (dMMR), microsatellite instability–high (MSI-H), and high tumor mutational burden (TMB) have provided the basis for PD-1 (PD-1; PDCD1) blockade across histologies and enabled tumor-agnostic indications (13–15). In ampullary carcinoma, several series report dMMR or MSI-H in a meaningful minority of cases, often in tumors with intestinal or medullary features and prominent lymphocytic infiltrates (16–18). National Comprehensive Cancer Network guidelines recommend routine mismatch repair (MMR) assessment in ampullary carcinoma and list checkpoint inhibitors among systemic options for appropriately selected advanced dMMR/MSI-H tumors, while noting that some evidence is extrapolated from related gastrointestinal (GI) primaries (8, 19).

Beyond MMR, the broader immune biology of ampullary carcinoma is not well defined. Expression of programmed cell death ligand 1 (PD-L1) in ampullary and periampullary cancers varies and is influenced by assay choice, scoring algorithm, and cutpoints. Consequently, the relationship between PD-L1 expression and outcome remains uncertain (20–22). Independent of PD-L1, immunophenotyping has defined ampullary carcinoma subgroups with distinct clinicopathologic behavior and generated subtype-specific treatment hypotheses (23). In periampullary cohorts, CD8-related immune composition and proximity to tumor cells have been associated with outcomes (20, 24), supporting compartment-aware analysis. Clinical experience with immune checkpoint blockade in ampullary carcinoma remains largely limited to case reports documenting both marked responses in biomarker-selected disease and hyperprogression (25, 26), underscoring the need for a more granular characterization of immune markers in this disease.

This study evaluated the prevalence and distribution of MMR status, PD-L1 expression, and compartment-specific CD3 and CD8 tumor-infiltrating lymphocytes (TIL) in resected ampullary adenocarcinomas and explored their associations with disease-specific survival (DSS). We also described a simple MMR/CD8 phenotype for future investigation. Because no patient received immune checkpoint blockade, all treatment-related interpretations were prespecified as hypothesis generating rather than predictive. This represents one of the larger ampullary-specific cohorts, in which MMR, PD-L1 combined positive score (CPS), and compartment-resolved CD3/CD8 infiltration have been evaluated together with long-term disease-specific outcomes.

## Materials and Methods

### Study cohort

This retrospective study included 99 consecutive patients who underwent pancreaticoduodenectomy for histologically confirmed ampullary adenocarcinoma at Vancouver General Hospital (VGH) between 1984 and 2013. Cases were identified from the pathology archives with clinicopathologic variables and outcomes abstracted from the electronic medical record. This cohort has been described previously (23). Pathologic stage was reviewed and harmonized to American Joint Committee on Cancer Seventh Edition criteria (27); nine original pTX entries underwent review of the original pathology material and reports. Cause-of-death classification followed the diagnosis recorded in the clinical record used for cohort abstraction; deaths not recorded as attributable to ampullary adenocarcinoma were censored. Postoperative mortality was not analyzed as a separate endpoint. Recurrence information was not sufficiently complete or standardized across the study interval to support recurrence-free survival analysis. Historical preoperative imaging and workup could not be reconstructed consistently and were not analyzed. The BC Cancer Research Ethics Board approved the study (H22-00073) and waived the requirement for written informed consent because the research used archived material and retrospective clinical data, as many patients were deceased and recontact would have been impracticable. The study was conducted in accordance with the Declaration of Helsinki and the Tri-Council Policy Statement v2: Ethical Conduct for Research Involving Humans.

### Histopathologic review and subtype classification

All cases underwent centralized review by one GI pathologist to confirm primary ampullary adenocarcinoma, assign histologic subtype using established morphologic criteria utilizing supporting immunohistochemistry (IHC) where needed (23, 28), and harmonized stage. Review was blinded to outcome. One tumor initially recorded as mixed was reclassified as PB according to its dominant morphology; the final cohort therefore comprised 47 PB and 52 intestinal tumors.

### IHC and TIL scoring

Duplicate 0.6-mm tissue microarray (TMA) cores were constructed from residual formalin-fixed, paraffin-embedded tumors. The following antibodies were used: MLH1 (clone ES05, Leica, NCL-L-MLH1, RRID: AB_10555424), PMS2 (EP51, Agilent, M3647, RRID: AB_3331634), MSH2 (G219-1129, Cell Marque, 286M-16, RRID: AB_1160596), MSH6 (EP49, Agilent, M3646, RRID: AB_3331636), CD3 (2GV6, Roche/Ventana, 790-4341, RRID: AB_2335978), CD8 (SP57, Roche/Ventana, 790-4460, RRID: AB_2335985), and PD-L1 (SP142, Abcam, ab228462, RRID: AB_2827816). MMR, CD3, and CD8 staining was performed using the Ventana platform in the Department of Anatomical Pathology at VGH; PD-L1 staining was performed at the Molecular and Cellular Immunology Core, Deeley Research Centre. dMMR was defined as complete loss of tumor nuclear staining for one or more MMR proteins with intact internal controls. PD-L1 was summarized using the CPSs, calculated as the number of PD-L1–staining tumor cells, lymphocytes, and macrophages divided by the total number of viable tumor cells and multiplied by 100. CPS ≥1 and CPS ≥10 were prespecified exploratory thresholds. Neither these cutpoints nor the research-use SP142/CPS combination has been clinically validated in ampullary adenocarcinoma. Absolute CD3^+^ and CD8^+^ cell counts were recorded separately in stromal and epithelial compartments and were not normalized to tissue area. Intraepithelial presence required at least one positive intraepithelial cell in either evaluable core; absence required zero across evaluable cores. The primary patient-level count was the maximum across duplicate cores to reduce false-negative classification of focal infiltrates; when one core was evaluable, that value was used. Cores with less than 10% residual tissue were excluded. No formal interobserver reproducibility study or whole-section validation was available.

### Biomarker summaries and sensitivity definitions

Biomarker prevalence, missingness, TIL localization, and count distributions were summarized using raw counts, percentages, medians, interquartile ranges (IQR), and empirical cumulative distribution functions. The primary TIL assessment metric was intraepithelial presence versus absence. Continuous sensitivity models used the natural logarithm (log) of the maximum-core count plus one, ln (count + 1). A duplicate-core mean, transformed as ln (mean + 1), assessed sensitivity to the maximum-core rule. Cohort-mean dichotomies were retained only as secondary sensitivity analyses; epithelial and stromal cutpoints were 11 and 56 cells for CD3 and 5 and 27 cells for CD8, respectively.

Mean-split categories were defined as above versus at or below the marker- and compartment-specific cohort mean. Because epithelial counts were zero-inflated and cohort means have limited clinical interpretability, these categories were not used for the primary CD8 analysis.

### Descriptive immune phenotypes

One descriptive phenotype scheme was used consistently: dMMR regardless of CD8 localization; inflamed proficient MMR (pMMR), defined as pMMR with intraepithelial CD8^+^ T-cell presence; and noninflamed pMMR, defined as pMMR without intraepithelial CD8^+^ T-cell presence. The scheme was intended to summarize biological patterns and generate trial-stratification hypotheses, not to define clinical actionability or predict checkpoint inhibitor benefit.

### Statistical analysis

DSS was measured as the time from pancreaticoduodenectomy to death recorded as attributable to ampullary adenocarcinoma; other deaths and patients alive at last follow-up were censored. The median follow-up was estimated using the reverse Kaplan–Meier method. Kaplan–Meier curves and log-rank tests summarized univariable DSS. Prespecified exploratory Cox models used complete cases and were adjusted for age at surgery (continuous), histologic subtype (PB vs. intestinal), grouped pathologic tumor (pT) stage (pT3–4 vs. pT1–2), pathologic node (pN) status (pN1 vs. pN0), and adjuvant chemotherapy (yes vs. no); pNX cases were excluded. Grade and margin status were summarized descriptively but not modeled because only two tumors were G1, five were G3, and six had an R1 margin. Each biomarker was evaluated in a separate model to limit model complexity. Associations were reported as hazard ratios (HR) with profile-likelihood 95% confidence intervals (CI) and likelihood-ratio *P* values. Wald tests were reported only as explicitly labeled sensitivity analyses. Continuous count models used ln (count + 1). Proportional hazards assumptions were assessed using standard diagnostics. Biomarker associations with subtype, grouped pT, and pN were assessed using likelihood-ratio tests. Analyses were exploratory, two-sided, and performed in JMP Pro version 18 (SAS Institute; RRID: SCR_022199).

## Results

The cohort included 99 patients and 44 DSS events. The median follow-up by reverse Kaplan–Meier estimation was 7.08 years (95% CI, 5.48–9.36). Clinicopathologic characteristics and assay-specific missingness are summarized in Table 1A and B. The final grouped pT distribution was 59 pT1 to pT2 tumors (59.6%) and 40 pT3 to pT4 tumors (40.4%). Margin status was R0 in 92, R1 in 6, and RX in 1. Histopathologic grade was available for 98 tumors (G1: *n* = 2; G2: *n* = 91; G3: *n* = 5).

**Table 1.** Cohort description.

A. Clinicopathologic characteristic	
Characteristic	Value
Age at surgery, years	69.5 (IQR, 59.2–75.6)
Sex, *n* (%)	Female: 43 (43.4%); male: 56 (56.6%)
Histologic subtype, *n* (%)	PB: 47 (47.5%); intestinal: 52 (52.5%)
Grouped pT stage, *n* (%)	pT1–2: 59 (59.6%); pT3–4: 40 (40.4%)
pN stage, *n* (%)	pN0: 49 (49.5%); pN1: 48 (48.5%); pNX: 2 (2%)
Histologic grade (evaluable *n* = 98), *n* (%)	G1: 2 (2%); G2: 91 (92.9%); G3: 5 (5.1%); missing: 1
Margin status, *n* (%)	R0: 92 (92.9%); R1: 6 (6.1%); RX: 1 (1%)
Adjuvant chemotherapy, *n* (%)	Yes: 15 (15.2%); no: 84 (84.8%)
DSS status, *n* (%)	Death from disease: 44 (44.4%); censored: 55 (55.6%)
Median follow-up, years	7.08 (reverse Kaplan–Meier 95% CI, 5.48–9.36)

B. Prevalence and distribution of immune biomarkers	
Biomarker	Value
MMR status (*n* = 99), *n* (%)	pMMR: 62 (62.6%); dMMR: 37 (37.4%)
PD-L1 CPS (evaluable *n* = 87; missing *n* = 12)	1 (IQR, 0–10)
PD-L1 CPS ≥1 (among evaluable)	57/87 (65.5%)
PD-L1 CPS ≥10 (among evaluable)	24/87 (27.6%)
CD3 localization (evaluable *n* = 97; missing *n* = 2)	Stroma only: 57 (58.8%); stroma and epithelium: 40 (41.2%)
CD8 localization (evaluable *n* = 98; missing *n* = 1)	Absent: 9 (9.2%); stroma only: 66 (67.3%); stroma and epithelium: 23 (23.5%)
CD3 stromal count	48 (IQR, 24–75)
CD3 epithelial count	0 (IQR, 0–7)
CD8 stromal count	18 (IQR, 5–39)
CD8 epithelial count	0 (IQR, 0–0)
CD3 mean-split sensitivity	Epithelial cutpoint: 11 (high 18/97); stromal cutpoint: 56 (high 41/97)
CD8 mean-split sensitivity	Epithelial cutpoint: 5 (high 17/98); stromal cutpoint: 27 (high 41/98)

**Figure 1.**
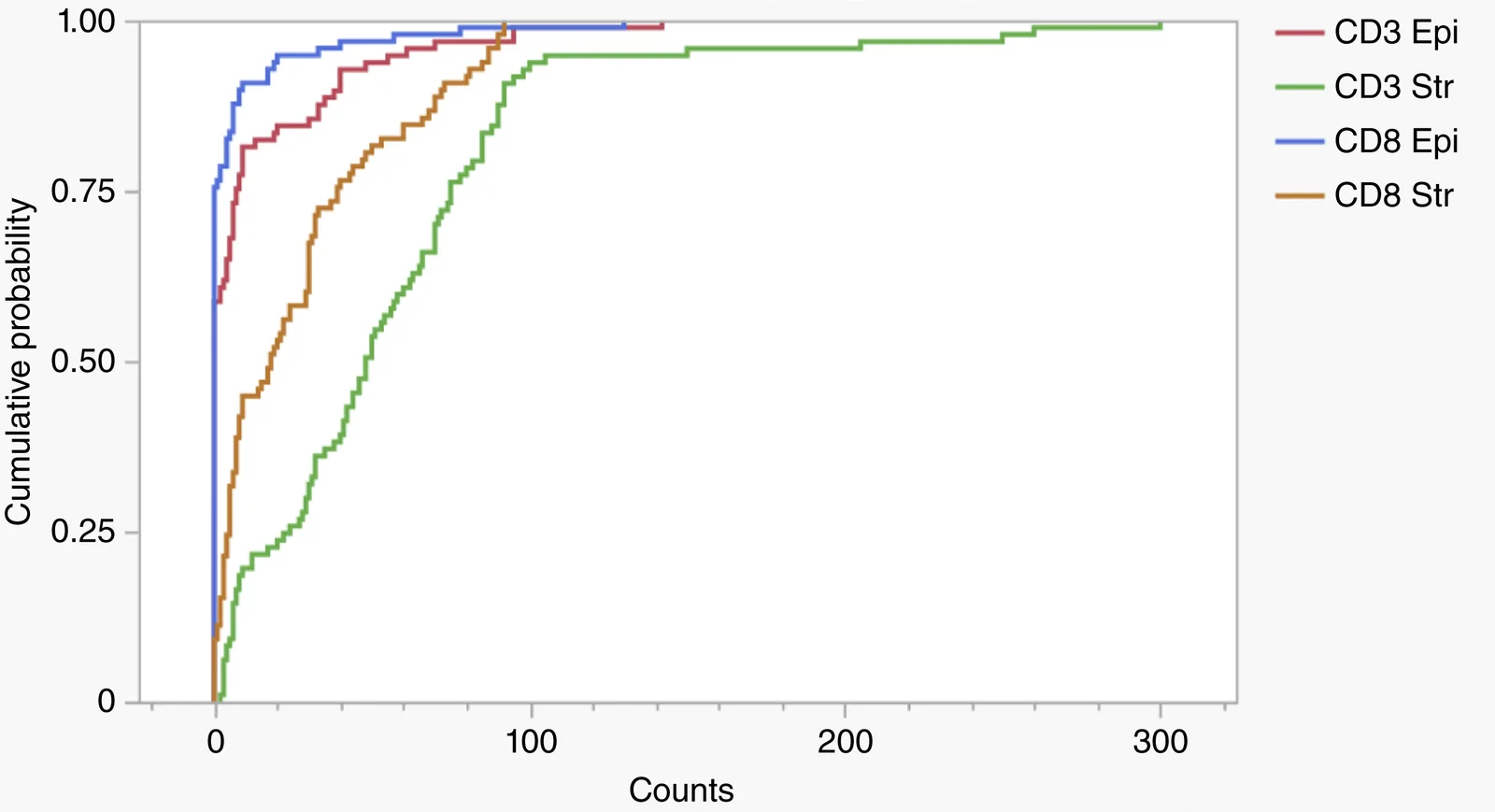
Empirical cumulative distribution functions of CD3^+^ and CD8^+^ TIL counts by compartment. Epi, epithelium; Str, stroma.

dMMR was present in 37 of 99 (37.4%) tumors. PD-L1 was evaluable in 87 tumors; the median CPS was 1 (IQR, 0–10), and CPS ≥1 occurred in 57 of 87 (65.5%) cases and CPS ≥10 in 24 of 87 (27.6%) cases. Intraepithelial CD3^+^ and CD8^+^ T cells were present in 40 of 97 (41.2%) and 23 of 98 (23.5%) tumors, respectively. Count distributions are shown in Fig. 1. The secondary mean-split categories classified 18 of 97 and 41 of 97 tumors as high for epithelial and stromal CD3 and 17 of 98 and 41 of 98 as high for epithelial and stromal CD8, respectively.

### Biomarker associations with clinicopathologic covariates

Biomarker–pathology associations are summarized in Table 2. Intraepithelial CD8 presence was more frequent in pT1 to pT2 than pT3 to pT4 tumors [20/59 (33.9%) vs. 3/39 (7.7%) among evaluable tumors; likelihood-ratio *P* = 0.0015] and showed a weaker association with pN status (*P* = 0.064). Intraepithelial CD3 presence was associated with histologic subtype (*P* = 0.013) and grouped pT (*P* = 0.046). MMR status and PD-L1 CPS ≥10 were not associated with grouped pT or pN.

**Table 2.** Likelihood-ratio *P* values for biomarker associations with pathology variables.

Biomarker	Histologic subtype	Grouped pT	pN status
MMR status	0.0645	0.9829	0.3579
PD-L1 CPS ≥10	0.5499	0.2926	0.4241
Intraepithelial CD8 presence	0.1453	0.0015	0.0643
Intraepithelial CD3 presence	0.0130	0.0462	0.2278

NOTE: *P* values are from likelihood-ratio tests. pNX cases were excluded from pN comparisons.

### Univariable DSS analyses

**Figure 2.**
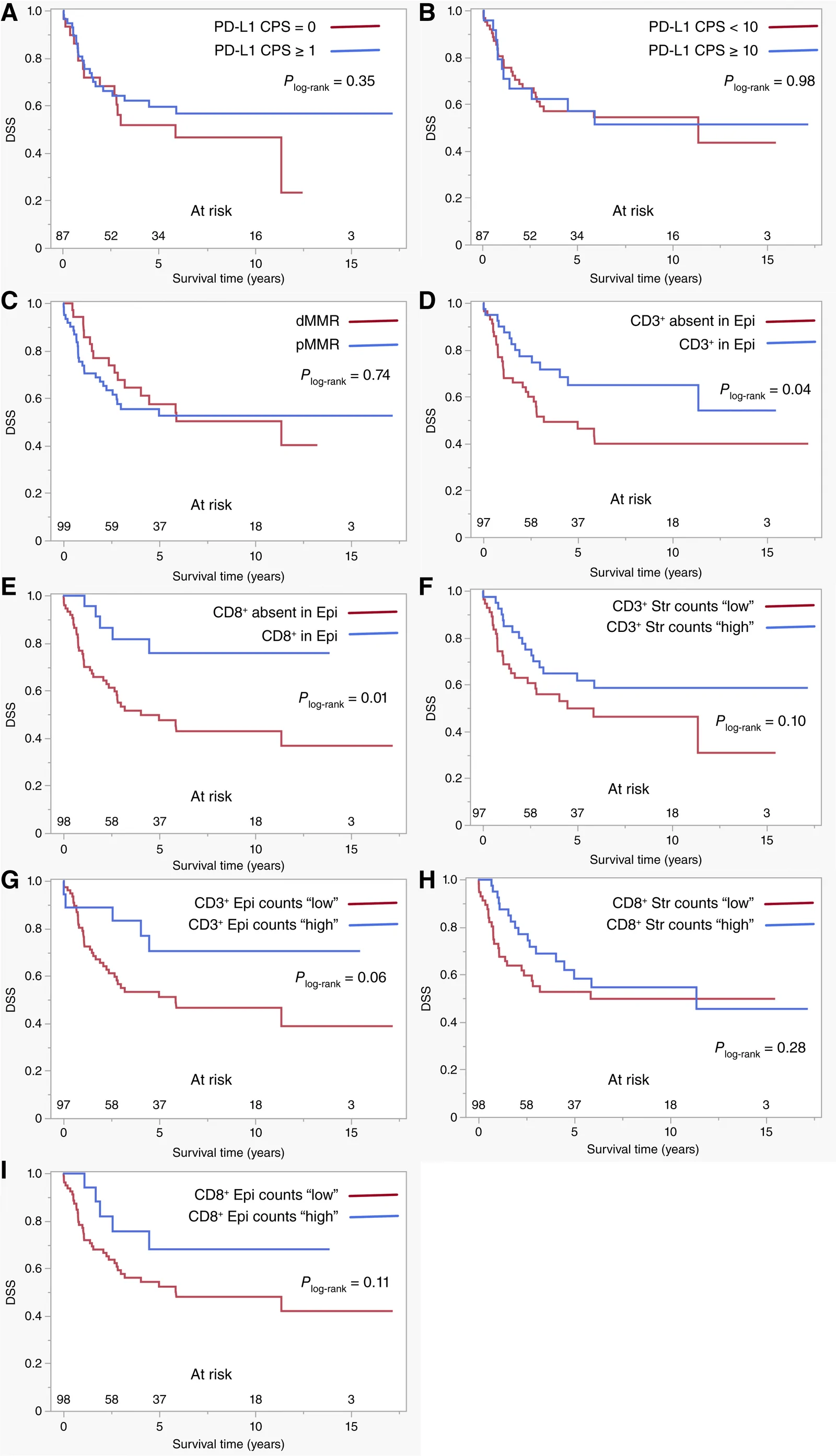
Univariable DSS according to immune biomarkers. Kaplan–Meier curves show DSS stratified by (**A**) PD-L1 CPS 0 vs. ≥1; (**B**) PD-L1 CPS <10 vs. ≥10; (**C**) dMMR vs. pMMR; (**D**) intraepithelial CD3^+^ T-cell presence vs. absence; (**E**) intraepithelial CD8^+^ T-cell presence vs. absence; (**F**) stromal CD3^+^ T-cell counts categorized as low vs. high; (**G**) intraepithelial CD3^+^ T-cell counts categorized as low vs. high; (**H**) stromal CD8^+^ T-cell counts categorized as low vs. high; and (**I**) intraepithelial CD8^+^ T-cell counts categorized as low vs. high. High vs. low count categories were defined using the cohort mean for each marker and compartment (CD3: 56 cells in stroma and 11 cells in epithelium; CD8: 27 cells in stroma and 5 cells in epithelium). Numbers at risk are displayed within each panel. *P* values are from two-sided log-rank tests. Epi, epithelium; Str, stroma.

PB subtype (HR, 2.02; profile-likelihood 95% CI, 1.11–3.76; likelihood-ratio *P* = 0.022), pT3 to pT4 stage (HR, 3.20; 95% CI, 1.75–6; *P* = 0.0002), and pN1 status (HR, 2.31; 95% CI, 1.26–4.36; *P* = 0.0069) were associated with inferior DSS. Adjuvant chemotherapy failed to demonstrate a significant impact on outcome (HR, 0.78; 95% CI, 0.30–1.72; *P* = 0.567). PD-L1 CPS ≥1, PD-L1 CPS ≥10, and MMR status were also not associated with DSS (log-rank *P* = 0.35, *P* = 0.98, and *P* = 0.74, respectively). Intraepithelial CD3 and CD8 presence showed univariable separation (*P* = 0.04 and *P* = 0.01, respectively; Fig. 2).

### Exploratory multivariable DSS analyses

The primary intraepithelial CD8 presence model included 96 patients and 43 DSS events after excluding pNX cases and adjusting for age, histologic subtype, grouped pT, pN, and adjuvant chemotherapy. Intraepithelial CD8 presence was associated with a lower disease-specific hazard (HR, 0.422; profile-likelihood 95% CI, 0.144–0.994; likelihood-ratio *P* = 0.048), but the corresponding Wald test was not significant (*P* = 0.074). Continuous natural log–transformed intraepithelial CD8 counts showed a concordant estimate (HR, 0.718 per natural-log unit; profile-likelihood 95% CI, 0.474–0.980; likelihood-ratio *P* = 0.035), with a nonsignificant Wald sensitivity test (*P* = 0.067). Continuous stromal CD8 and stromal or epithelial CD3 counts were not associated with DSS (Table 3). Using the duplicate-core mean rather than maximum produced a similar intraepithelial CD8 estimate (HR, 0.714; profile-likelihood 95% CI, 0.464–0.986; likelihood-ratio *P* = 0.040; Wald *P* = 0.072). All four mean-split TIL sensitivity models were nonsignificant (Wald *P* = 0.079–0.239). Additional adjustment for MMR status did not materially change the direction of the CD8 presence estimate. Because likelihood-ratio and Wald inferences differed, the CD8 finding was considered borderline and method dependent rather than independently established.

**Table 3.** Exploratory adjusted associations between TIL measures and DSS.

Biomarker exposure	*n* (events)	Adjusted HR	Profile 95% CI	Likelihood-ratio *P*
Intraepithelial CD8 presence (present vs. absent)	96 (43)	0.422	0.144–0.994	0.048
ln (CD3 stromal count + 1), per natural-log unit	95 (43)	0.835	0.627–1.122	0.227
ln (CD3 epithelial count + 1), per natural-log unit	95 (43)	0.810	0.625–1.017	0.070
ln (CD8 stromal count + 1), per natural-log unit	96 (43)	0.953	0.751–1.214	0.695
ln (CD8 epithelial count + 1), per natural-log unit	96 (43)	0.718	0.474–0.980	0.035

NOTE: Each row is a separate complete-case Cox model adjusted for age, histologic subtype, grouped pT stage, pN status, and adjuvant chemotherapy. pNX cases (*n* = 2) were excluded. Counts use the maximum evaluable TMA core. CI and *P* values are profile-likelihood and likelihood-ratio estimates. The Wald sensitivity *P* values were 0.074 for CD8 presence and 0.067 for continuous epithelial CD8.

### Descriptive MMR/CD8 phenotypes

**Figure 3.**
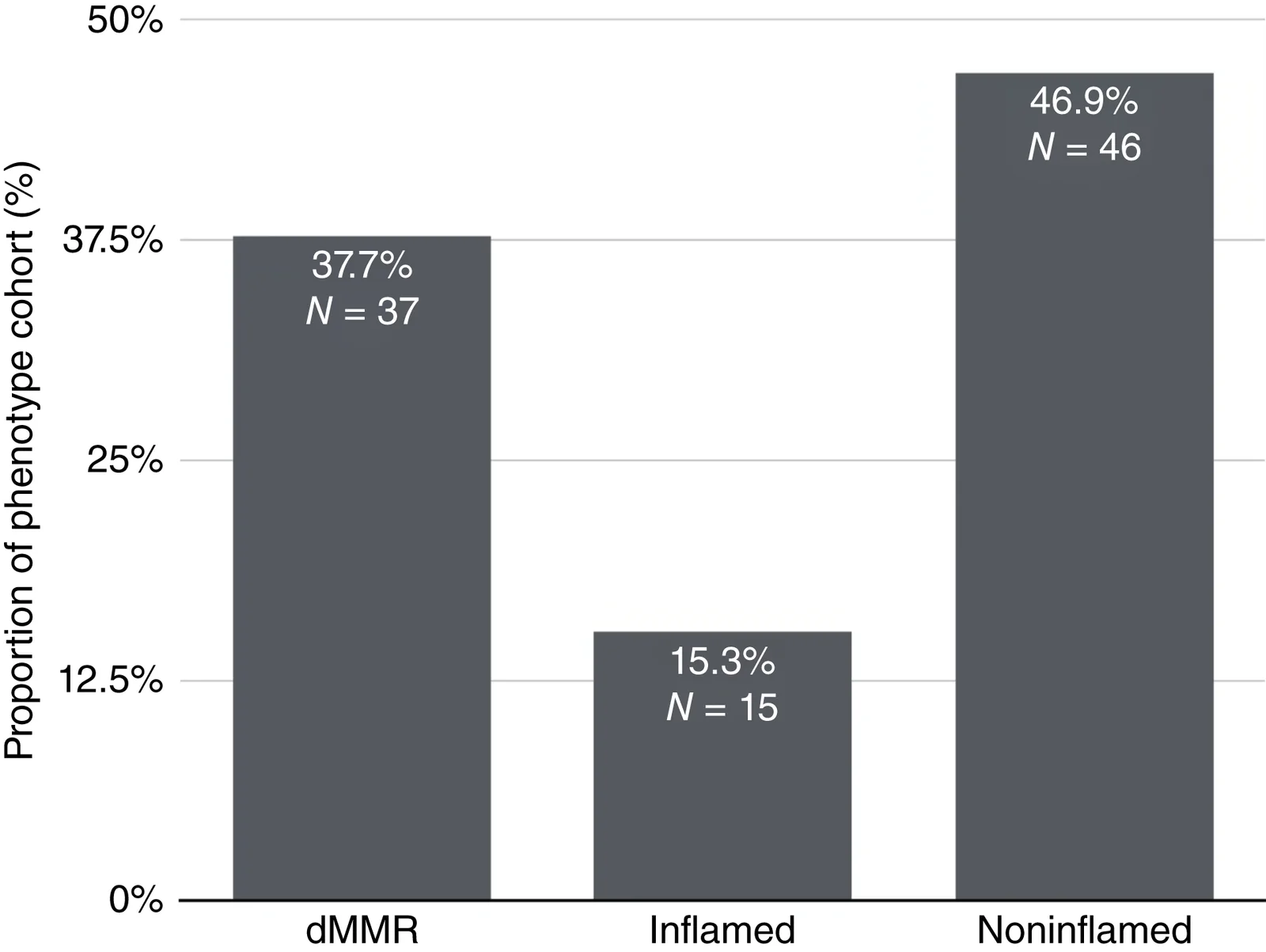
Prevalence of descriptive MMR/CD8 phenotypes among 98 tumors with both markers evaluable. dMMR was assigned regardless of CD8 localization; inflamed pMMR denotes pMMR with intraepithelial CD8^+^ T-cell presence; and noninflamed pMMR denotes pMMR without intraepithelial CD8^+^ T-cell presence. The categories are descriptive and are not treatment-selection classes.

Among 98 tumors with both MMR and epithelial CD8 data, 37 (37.7%) were dMMR, 15 (15.3%) were inflamed pMMR, and 46 (46.9%) were noninflamed pMMR (Fig. 3). The phenotype was used only as a descriptive summary. No survival or treatment-predictive claim was made for these small groups.

## Discussion

The principal observation of this study was compartment specificity. Intraepithelial, but not stromal, CD8 infiltration showed a concordant favorable association with DSS across presence-based, continuous maximum-core, and duplicate-core mean analyses after adjustment for stage and other clinical covariates. These findings suggest that future ampullary biomarker studies should preserve spatial compartment information because total or stromal lymphocyte measurements may obscure a potentially prognostic epithelial immune signal. In this retrospective resection cohort, dMMR and PD-L1 expression were common, whereas spatial localization of CD8^+^ T cells showed the clearest prognostic signal. After adjustment including grouped pT, intraepithelial CD8 presence and continuous intraepithelial CD8 count both favored lower disease-specific hazard, but likelihood-ratio and Wald inferences differed. The result is therefore best regarded as a borderline, hypothesis-generating association that requires external validation rather than as an established independent prognostic or predictive marker.

The frequency of dMMR was higher than that in prior ampullary and periampullary series (16–18). This difference may reflect cohort composition, assay methodology, or sampling and should be examined in external cohorts. dMMR remains the most directly clinically relevant finding because its relationship to checkpoint blockade is supported by tumor-agnostic evidence and guideline recommendations (8, 13, 29). Our cohort did not include immunotherapy-treated patients, so it provides no new estimate of treatment benefit. Functional MSI testing and TMB were not available; these omissions prevent molecular confirmation and assessment of additionally approved biomarker categories.

PD-L1 expression was frequent but not prognostic at CPS ≥1 or CPS ≥10. This is consistent with the assay, scoring, and cutpoint-dependent literature in ampullary and related GI cancers (20–22). The absence of a DSS association should not be interpreted as evidence for or against response to PD-1/PD-L1 inhibition.

The concordant direction of the presence/absence, continuous maximum-core, and duplicate-core mean analyses supports further study of intraepithelial CD8 localization. Recent single-cell transcriptomic and spatial proteomic profiling of three intestinal-type ampullary carcinomas also identified an immunosuppressive tumor immune microenvironment, illustrating the value of higher-resolution immune mapping; the sample was too small to support clinical stratification (30). However, duplicate 0.6-mm TMA cores incompletely sampled spatial heterogeneity; maximum-core scoring can inflate counts in focally inflamed tumors; counts were not area-normalized; and neither interobserver reproducibility nor whole-section validation was available. These constraints are important because the observed statistical signal was borderline and sensitive to the inferential method.

The three-group MMR/CD8 phenotype offers a testable framework for prospective therapeutic trial stratification. The presence of 15 tumors meeting the inflamed pMMR definition demonstrates that pMMR ampullary adenocarcinoma is not uniform with respect to epithelial CD8 engagement; a subset contains intraepithelial CD8^+^ T cells, whereas others do not. This heterogeneity may be obscured when all pMMR tumors are analyzed as a single category. The framework therefore supports prospective trials that prespecify inflamed and noninflamed pMMR strata and formally test whether treatment effects differ between them. Conceptually, it enables two distinct therapeutic questions of whether checkpoint blockade has differential activity when epithelial CD8 engagement is already present and whether tumors lacking epithelial CD8 require strategies that improve immune priming or recruitment before checkpoint release (31). These are mechanistic and trial design hypotheses, not treatment recommendations. No patient in this cohort received checkpoint blockade, the pMMR subgroups were small, and no biomarker-by-treatment interaction could be estimated. Any predictive claim will require standardized compartment-specific assessment and an interaction-based analysis in an immunotherapy-treated cohort.

Additional limitations include the single-center retrospective design, 44 disease-specific events, multiple exploratory comparisons, sparse grade and positive-margin categories, assay-specific missingness, incomplete recurrence ascertainment, and the 1984 to 2013 treatment era. Only 15 patients received adjuvant chemotherapy, limiting contemporary generalizability. Year of surgery was not associated with DSS in the sensitivity analysis (likelihood-ratio *P* = 0.914), and no temporal association was detected for PD-L1 CPS ≥10, CD3 localization, CD8 localization, or MMR status (all *P* ≥ 0.67); these findings reduce but do not eliminate concern about archival tissue and era effects. Prospective, multi-institutional validation with whole sections or spatial profiling, molecular correlates, standardized outcomes, and treatment exposure is needed.

In summary, immune biomarkers are heterogeneous in resected ampullary adenocarcinoma. dMMR was common and PD-L1 was not prognostic, whereas intraepithelial CD8 showed a cautious, method-dependent association with DSS. The principal translational observation is that pMMR ampullary adenocarcinoma is not a single immune state; intraepithelial CD8 identifies a descriptive subset with epithelial immune engagement that should be distinguished prospectively from a noninflamed pMMR disease. These findings support external prognostic validation and incorporation of standardized spatial CD8 assessment into therapeutic trial stratification; they do not justify treatment selection or extension of checkpoint blockade beyond established biomarker indications.

## Data Availability

Deidentified record-level data are not publicly available because ethical approval did not include public sharing, and clinicopathologic data from this rare disease may remain reidentifiable. Qualified researchers may request access from the corresponding author, subject to institutional and research ethics approval and an appropriate data-sharing agreement.
